# Emerging targeted therapies for chronic obstructive pulmonary disease: from symptom control to precision medicine

**DOI:** 10.3389/fphar.2026.1911892

**Published:** 2026-08-24

**Authors:** Guangwei Chen, Haishun Yang, Weihang Sun, Jiawen Shen, Fang Ma, Jinli Qin, Xianhong Wang, Yalong Liu, Na Chen, Tieshan Teng, Shichang Sun, Xinying Ji

**Affiliations:** 1 Pulmonology Department, Fangcheng County People’s Hospital, Nanyang, Henan, China; 2 Henan International Joint Laboratory for Nuclear Protein Regulation, School of Basic Medical Sciences, Henan University, Kaifeng, Henan, China; 3 Department of Microbiology and Immunology, Henan Provincial Research Center of Engineering Technology for Nuclear Protein Medical Detection, School of Medicine and Health Sciences, Zhengzhou Health College, Zhengzhou, Henan, China; 4 Department of Pulmonary and Critical Care Medicine, the First Affiliated Hospital of Henan University, Kaifeng, China; 5 Department of Pulmonary and Critical Care Medicine, Huaihe Hospital of Henan University, Henan University, Kaifeng, China

**Keywords:** chronic obstructive pulmonary disease (COPD), mesenchymal stem cells, monoclonal antibodies, Nrf2–Keap1 pathway, precision therapy

## Abstract

Chronic obstructive pulmonary disease (COPD) is a highly prevalent chronic inflammatory disorder of the airways characterized by substantial morbidity, disability, and mortality. It poses a significant global public health burden. Conventional therapies for COPD, which mainly rely on bronchodilators and inhaled corticosteroids, effectively relieve symptoms and reduce exacerbation frequency in patients. However, their clinical utility is limited by safety concerns and heterogeneous treatment responses across disease phenotypes. Moreover, these therapeutic approaches cannot halt or reverse disease progression. With increasing understanding of the complex molecular and cellular mechanisms underlying COPD pathogenesis, therapeutic approaches are evolving from conventional symptom-oriented management toward precision medicine and mechanism-based targeted interventions. This review systematically summarizes recent advances in emerging targeted therapies for COPD, including monoclonal antibodies directed against key inflammatory pathways, small-molecule modulators targeting core pathogenic mechanisms, and stem cell-based therapies. By selectively modulating critical processes involved in chronic inflammation, tissue injury, and airway remodeling, these novel therapeutic strategies offer promising therapeutic opportunities, particularly for patients with corticosteroid resistance and frequent exacerbations.

## 1 Introduction

COPD is a progressive respiratory disorder characterized by persistent airflow limitation and complex pathophysiological mechanisms, including small airway dysfunction, mucus hypersecretion, chronic inflammation, oxidative stress, airway remodeling, and corticosteroid resistance. As the third leading cause of death worldwide, COPD imposes a substantial global public health burden (Han et al., 2021; Agustí et al., 2023). Current pharmacological management is primarily aimed at relieving airflow limitation, suppressing airway inflammation, reducing acute exacerbations, and slowing lung function decline. Inhaled corticosteroids (ICS) and bronchodilators are standard therapeutic agents routinely used in clinical practice, with the latter including long-acting β2-adrenergic agonists (LABA) and long-acting anticholinergic agents (LAMA) (Buhl et al., 2020; Martinez et al., 2020).

Although the aforementioned therapies for COPD are extensively employed in clinical practice, their inherent limitations are equally apparent and are not to be disregarded. First of all, ICS-containing regimens are associated with an increased risk of pneumonia, and their potential relationship with cardiovascular adverse events remains under debate, necessitating careful assessment of the benefit–risk profile (Lipson et al., 2018; Rabe et al., 2020; Martinez et al., 2021; Ioannides et al., 2025). In addition, treatment responses vary considerably among clinical phenotypes, and current therapies are often inadequate in patients with corticosteroid resistance or frequent exacerbations. More importantly, these therapeutic modalities are insufficient to directly intervene in disease progression and thus reverse lung function decline (van der Koog et al., 2026). As a consequence, the development of targeted drugs for COPD has become imperative. Recent advances in elucidating the molecular mechanisms underlying COPD progression are catalyzing a paradigm shift from symptom-oriented therapy toward precision targeted medicine (Kelly et al., 2025; Ahmadi et al., 2023; Gao et al., 2025). This review provides a comprehensive overview of recent advances in three major categories of emerging therapies for COPD: monoclonal antibodies targeting inflammatory pathways, small-molecule modulators of core pathogenic mechanisms, and stem cell-based therapies, with the aim of informing future clinical practice and therapeutic development.

## 2 Monoclonal antibody therapies

With increasing understanding of the inflammatory mechanisms underlying COPD, monoclonal antibody–based biologics have emerged as a promising strategy for precision therapy. By selectively neutralizing key cytokines or their receptors, these agents modulate specific inflammatory pathways and overcome the non-specific anti-inflammatory effects and limited efficacy of conventional therapies, particularly in patients with poor response to standard treatment. To date, two monoclonal antibodies, dupilumab and mepolizumab (Sciurba et al., 2025), have been approved for the treatment of COPD, demonstrating the translational value for inflammation-targeted therapy in this disease (Halpin et al., 2026; Bhatt et al., 2023; Ross et al., 2026). In addition, several emerging biologics are currently undergoing clinical evaluation, including tezepelumab, which targets thymic stromal lymphopoietin (TSLP), and itepekimab, tozorakimab, and astegolimab, which target the IL-33/ST2 signaling pathway. By modulating distinct inflammatory endotypes and their associated signaling networks, these agents have the potential to further expand the applicability of precision medicine in COPD and provide greater clinical benefits for selected patient subgroups (Calderon et al., 2023; Riera-Martínez et al., 2023).

### 2.1 Targeting TSLP in COPD

TSLP is an epithelial cell–derived alarmin that plays a key role in the initiation and amplification of both type 2 and non–type 2 airway inflammation. In patients with COPD, TSLP expression is markedly upregulated in the bronchial epithelium, bronchial mucosa, and airway smooth muscle, with expression levels increased by more than twofold compared with healthy controls (Anzalone et al., 2018). Structural, biophysical, and cellular analyses reveal that TSLP binds specifically to TSLPR, inducing allosteric activation through conformational remodeling of the αA helix. Activated TSLP subsequently recruits the shared receptor IL-7Rα, resulting in the formation of a functional TSLP–TSLPR–IL-7Rα ternary signaling complex (Verstraete et al., 2017). This signaling complex promotes the recruitment and activation of both eosinophils and neutrophils, contributing to the development of type 2 and non–type 2 inflammation in COPD. Furthermore, it directly stimulates airway smooth muscle cell proliferation and migration, thereby facilitating airway remodeling and disease progression.

Tezepelumab is a humanized IgG2λ monoclonal antibody that targets TSLP. By binding soluble TSLP, tezepelumab prevents the assembly of the TSLP–TSLPR–IL-7Rα signaling complex and thereby inhibits downstream inflammatory signaling pathways (Verstraete et al., 2017). Preclinical studies have indicated that tezepelumab exerts broad immunomodulatory effects by inhibiting the activation of dendritic cells, type 2 innate lymphoid cells, mast cells, eosinophils, and neutrophils. Furthermore, blockade of TSLP signaling suppresses the production of multiple downstream inflammatory mediators, including IL-4, IL-5, IL-13, and TNF-α, thereby attenuating both type 2 and non–type 2 airway inflammation (Verstraete et al., 2017). In the phase IIa COURSE trial, a randomized design was used to assign patients with moderate-to-severe COPD to receive tezepelumab (420 mg subcutaneously) or placebo for 52 weeks. Compared with placebo, tezepelumab showed a trend toward reducing the annualized rates of moderate-to-severe exacerbations (1.75 vs. 2.11) and severe exacerbations (0.13 vs. 0.25). Treatment also improved the CAT score by 1.86 points and reduced blood eosinophil counts and fractional exhaled nitric oxide (FeNO) levels by 71.3 cells/μL and 4.8 ppb, respectively, indicating favorable effects on airway inflammatory biomarkers (Singh et al., 2025a; Singh et al., 2025c).

Final analysis of the PASSAGE study—a Phase IV, single-arm, open-label, real-world clinical trial conducted in U.S. patients with severe uncontrolled asthma (SUA)—demonstrated that tezepelumab confers robust efficacy across a broad and heterogeneous patient population, encompassing subgroups historically underrepresented or systematically excluded from conventional randomized controlled trials (Lugogo et al., 2025; Lugogo et al., 2026). Of particular clinical relevance are the findings in two challenging subgroups: patients with asthma–COPD overlap (ACO), defined as severe asthma comorbid with mild-to-moderate chronic obstructive pulmonary disease, and individuals with a smoking history of ≥10 pack-years (Lugogo et al., 2026). In these cohorts, tezepelumab treatment was associated with a 65% and 64% reduction in annualized asthma exacerbation rates relative to baseline, respectively. Concurrently, both subgroups achieved clinically meaningful improvements in lung function, asthma control, and health-related quality of life, with all changes meeting or exceeding the established minimal clinically important difference thresholds. Given that tezepelumab acts upstream of type 2 inflammat ion through TSLP inhibition, independent of eosinophilic phenotype, it represents a novel pharmacological strategy for patients who are inadequately responsive to existing biologics—a mechanistic distinction that underscores the clinical translatability of these findings (Menzies-Gow et al., 2021).

### 2.2 Targeting IL-33 in COPD

Interleukin-33 (IL-33) is an epithelial cell–derived alarmin that is rapidly released from the nucleus into the extracellular milieu following cellular injury or environmental stimulation (Huang et al., 2021). Extracellular IL-33 binds to its specific receptor, suppression of tumorigenicity 2 (ST2), and activates downstream signaling pathways that amplify airway inflammation. In COPD, aberrant activation of the IL-33/ST2 axis contributes to both type 2 and non–type 2 inflammation and promotes fibroblast proliferation and collagen deposition, thereby facilitating airway remodeling (Kearley et al., 2015; Rabe et al., 2021). Human genetic evidence has provided strong support for targeting IL-33 in COPD. A genetic association study conducted by regeneron pharmaceuticals showed that loss-of-function variants of IL-33 reduced circulating IL-33 levels by 46% and were associated with a 21% lower risk of COPD. Conversely, gain-of-function variants increased susceptibility to the disease. These findings highlight the pathogenic role of IL-33 in COPD and provide a robust genetic rationale for the development of IL-33–targeted therapeutic strategies (Rabe et al., 2021).

Itepekimab is a high-affinity fully human IgG4 monoclonal antibody. Structural analyses by X-ray crystallography and cryo-electron microscopy revealed that its primary mechanism of action involves binding to the auxiliary Site 2 epitope of IL-33, resulting in partial inhibition of ST2 engagement (approximately 60%–70%) and attenuation of IL-33/ST2 signaling). In a phase IIa clinical trial, itepekimab has been reported to have biological activity against type 2 inflammatory pathways in COPD patients. Treatment reduced peripheral blood eosinophil counts by a mean of 20% at week 4 and 24% at the end of treatment in the overall population (Rabe et al., 2021). In the overall population, itepekimab reduced the annualized rate of moderate-to-severe exacerbations from 1.61 to 1.30, representing a 19% relative risk reduction, and improved FEV_1_ by 0.06 L from baseline between weeks 16 and 24. Notably, the therapeutic benefit was more pronounced in former smokers, with annualized moderate-to-severe exacerbation rates decreasing from 1.70 to 0.89 and severe exacerbation rates declining from 0.36 to 0.08. In addition, FEV_1_ increased by 0.09 L relative to baseline in this subgroup (Rabe et al., 2021). In subsequent phase III studies, itepekimab reduced the annualized exacerbation rate in patients with baseline blood eosinophil counts (BEC) ≥150 cells/μL, with annualized exacerbation rates of 1.52 and 2.40 in the itepekimab and placebo groups, respectively, corresponding to a 37% reduction in exacerbation risk. In the subgroup with BEC ≥300 cells/μL, itepekimab improved FEV_1_ by 0.146 L and reduced St. George’s Respiratory Questionnaire (SGRQ) scores by 9.53 points compared with placebo, indicating clinically meaningful improvements in lung function and health-related quality of life (Rabe et al., 2024; Baraldi et al., 2025; Sun, 2025).

Notwithstanding the robust genetic rationale and the encouraging early-phase clinical signals described above, the two pivotal Phase III trials of itepekimab in COPD—AERIFY-1 and AERIFY-2—yielded discordant outcomes (Rabe et al., 2021; Rabe et al., 2024). AERIFY-1 met its primary endpoint, demonstrating significant reductions in the annualized rate of moderate-to-severe exacerbations of 27.1% and 20.5% for the biweekly and every-4-week dosing regimens, respectively. By contrast, AERIFY-2 failed to corroborate these findings, with corresponding reductions of only 1.6% and 12.4% (neither statistically significant); moreover, the 24-week efficacy estimates (18% and 21%) were not sustained through 52 weeks. In both studies, the observed exacerbation event counts fell below projections, likely reflecting the confounding effects of COVID-19 pandemic-related protective measures, which may have attenuated the overall event rate and thereby compromised statistical power. To address these discrepancies, the sponsor (Sanofi/Regeneron) is currently conducting a comprehensive data review and consulting with regulatory agencies. Given the failure of AERIFY-2, the regulatory future of itepekimab remains highly uncertain, and its ultimate approval will hinge on further analyses and potential confirmatory evidence.

### 2.3 Tozorakimab in COPD

Tozorakimab is a fully human IgG1κ monoclonal antibody designed to neutralize both biologically active forms of IL-33. Strickson and colleagues reported that tozorakimab binds Site 1, the principal receptor-binding interface of IL-33, with sub-picomolar affinity. By selectively engaging reduced IL-33, it completely blocks the interaction between IL-33 and ST2, thereby preventing activation of the MyD88-dependent NF-κB and MAPK signaling cascades and subsequent production of pro-inflammatory cytokines. Notably, tozorakimab also recognizes oxidized IL-33 and inhibits IL-33-driven RAGE/EGFR signaling, a pathway implicated in epithelial dysfunction and mucus hypersecretion. Consequently, tozorakimab exerts both anti-inflammatory and epithelial-protective effects, reducing airway mucus overproduction while facilitating epithelial repair and restoration of airway homeostasis (England et al., 2023; Chen J. et al., 2026).

In a preclinical COPD mouse model, Rees and colleagues showed that tozorakimab provided substantial protection against structural lung injury. Treatment markedly attenuated airway smooth muscle thickening, peribronchial collagen deposition, and emphysema-associated alveolar destruction, as reflected by a reduction in mean linear intercept (MLI), indicating inhibition of airway remodeling and emphysema progression (England et al., 2023). In a phase I clinical study, Reid *et al.* reported that a 300 mg dose of tozorakimab exhibited potent anti-inflammatory activity *in vivo*. Treatment resulted in maximal reductions of 60% and 55% in serum IL-5 and IL-13 levels, respectively, together with a 42% decrease in peripheral blood eosinophil counts relative to baseline. These findings provide evidence that tozorakimab effectively suppresses type 2 inflammatory pathways and may contribute to the attenuation of airway inflammation in COPD (Reid et al., 2024). Results from a phase IIa clinical trial showed that, after 12 weeks of treatment, patients with moderate-to-severe COPD receiving tozorakimab had a 67-mL improvement in post-bronchodilator FEV_1_ relative to the placebo group. Notably, this benefit was sustained through week 28, extending beyond the active treatment period. In addition, tozorakimab reduced the risk of composite exacerbation events by 21% relative to placebo. In a predefined high-risk subgroup with a history of at least two exacerbations in the previous year, treatment with Tozorakimab resulted in an even greater improvement in post-bronchodilator FEV_1_, with a placebo-adjusted increase of 124 mL after 12 weeks, further supporting the potential therapeutic value of tozorakimab in high-risk patients with COPD (Singh et al., 2025b). Importantly, the therapeutic efficacy of tozorakimab was independent of smoking status, with comparable clinical benefits observed in both current and former smokers. This finding distinguishes tozorakimab from itepekimab, another IL-33–targeted therapy whose clinical efficacy has been primarily observed in former smokers, thereby overcoming an important limitation associated with this therapeutic class (Singh et al., 2025b). In Phase III MIRANDA, tozorakimab (300 mg every 2 weeks) added to inhaled maintenance therapy significantly reduced annualized moderate-to-severe COPD exacerbations in 1,450 symptomatic patients with persistent exacerbations despite therapy. The primary endpoint was met in former smokers and the overall population, with statistically and clinically significant reductions, and subgroup benefits were consistent across eosinophil counts and lung function severity.

### 2.4 Targeting ST2 in COPD

Astegolimab is a fully human IgG2 monoclonal antibody that selectively binds the ST2 receptor with high affinity. By preventing the interaction between IL-33 and ST2, it inhibits MyD88-dependent NF-κB signaling and downstream inflammatory responses, thereby attenuating IL-33-mediated airway inflammation and disease progression (Yousuf et al., 2022; Riera-Martínez et al., 2023). It has been reported that circulating sST2 levels were elevated in patients with COPD and were associated with disease severity, acute exacerbations, and lung function decline. Furthermore, sST2 was identified as an independent predictor of all-cause mortality, supporting the pathogenic and prognostic significance of ST2 and providing a clinical rationale for ST2-targeted therapy in COPD (Urban et al., 2021). In a phase II trial of patients with moderate-to-very severe COPD, the most favorable clinical outcomes were observed with the 490 mg regimen of astegolimab. Astegolimab reduced the annualized rate of moderate-to-severe exacerbations from 2.81 in the placebo group to 2.18, representing a 22% relative risk reduction. Moreover, treatment was associated with a 3.3-point reduction in SGRQ scores and a 40 mL increase in FEV_1_ compared with placebo, indicating clinically meaningful improvements in health-related quality of life and lung function, while also reducing the risk of disease exacerbations (Yousuf et al., 2022). Notably, astegolimab reduced peripheral blood and sputum eosinophil counts by 41% and 75%, respectively, supporting the role of IL-33/ST2 blockade in attenuating eosinophilic airway inflammation in COPD. Importantly, astegolimab was well tolerated, with comparable rates of treatment-related and serious adverse events between treatment groups and no treatment-related deaths (Yousuf et al., 2022).

**FIGURE 1:**
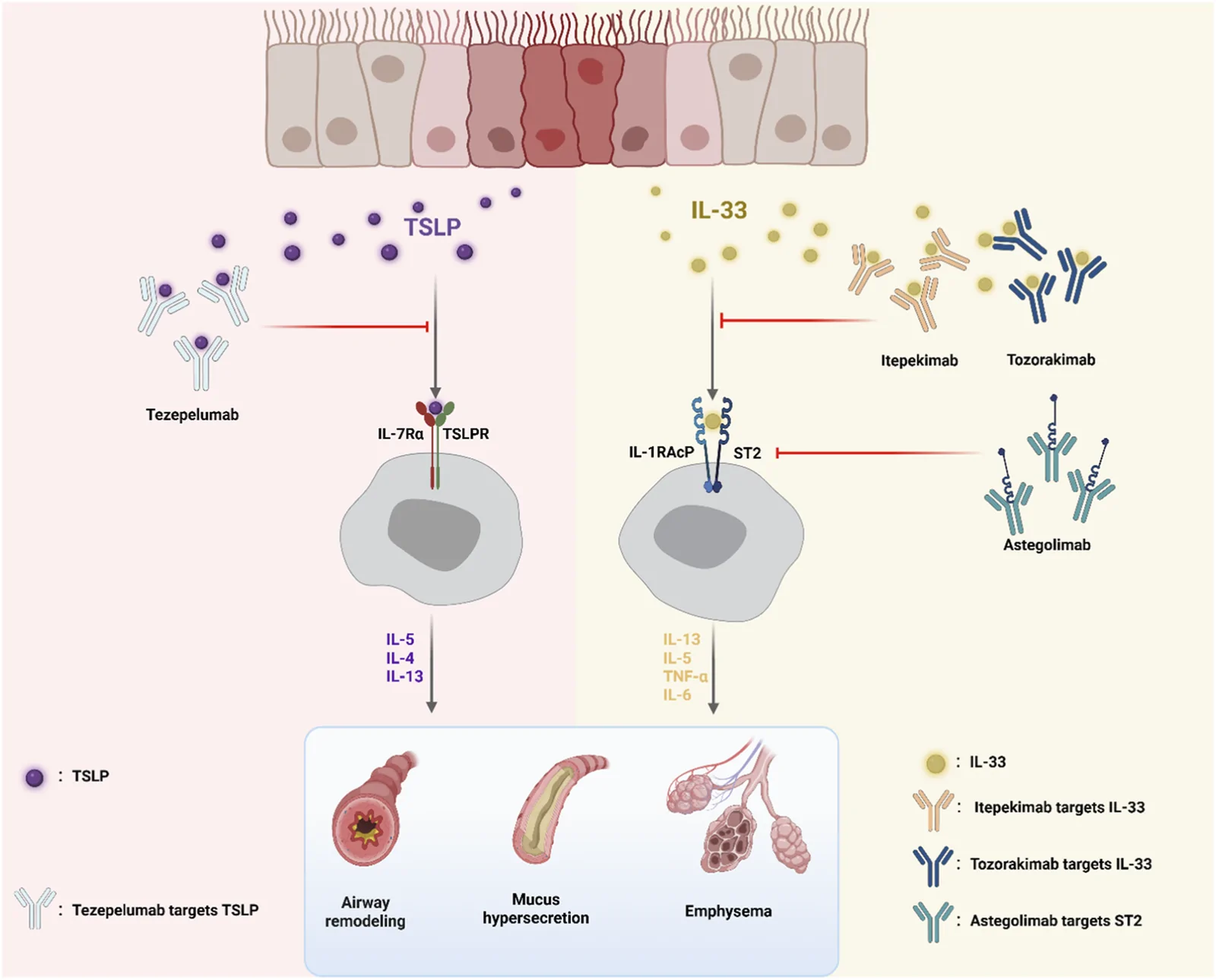
Therapeutic targeting of the TSLP/IL-33 axis in COPD. Epithelial-derived alarmins TSLP and IL-33 initiate upstream inflammatory signaling through the TSLPR–IL-7Rα and ST2–IL-1RAcP receptor complexes, respectively. Tezepelumab blocks TSLP, whereas itepekimab and tozorakimab neutralize IL-33. Astegolimab inhibits IL-33 signaling by targeting the ST2 receptor. Blockade of these pathways reduces downstream cytokine production and attenuates airway inflammation, mucus hypersecretion, airway remodeling, and emphysema, highlighting the therapeutic potential of alarmin-targeted biologics in COPD. This figure was enhanced using ChatGPT (GPT-4o) for resolution improvement and noise reduction.

The clinical benefits of astegolimab were further validated in the phase III ALIENTO and ARNASA trials. Studies have reported that, compared with placebo, astegolimab administered every 2 weeks reduced the risk of moderate-to-severe exacerbations by 15%, with a greater reduction of 22% observed among patients with a history of frequent exacerbations. In addition, the risks of severe exacerbations were reduced by 29% and 33% in the ALIENTO and ARNASA trials, respectively. Collectively, these findings provide further evidence supporting ST2/IL-33 pathway blockade as a promising therapeutic strategy for patients with exacerbation-prone COPD (Kelsen et al., 2025; Papi et al., 2026) (Figure 1).

## 3 Small-molecule regulators targeting pathological pathways of COPD

Small-molecule modulators have emerged as promising therapeutic agents for COPD by targeting key molecular mediators and signaling pathways involved in disease pathogenesis. Among them, p38 MAPK inhibitors suppress aberrant inflammatory signaling, whereas Nrf2 activators enhance endogenous antioxidant defenses, representing two of the most extensively investigated small-molecule strategies (Li et al., 2022). By targeting intracellular pathways and modulating complex signaling networks, these agents may overcome some limitations of monoclonal antibodies and provide new therapeutic options for patients with corticosteroid-resistant disease, frequent exacerbations, or oxidative stress–driven COPD phenotypes (Li et al., 2022).

### 3.1 Targeting the p38 MAPK signaling pathway

**FIGURE 2:**
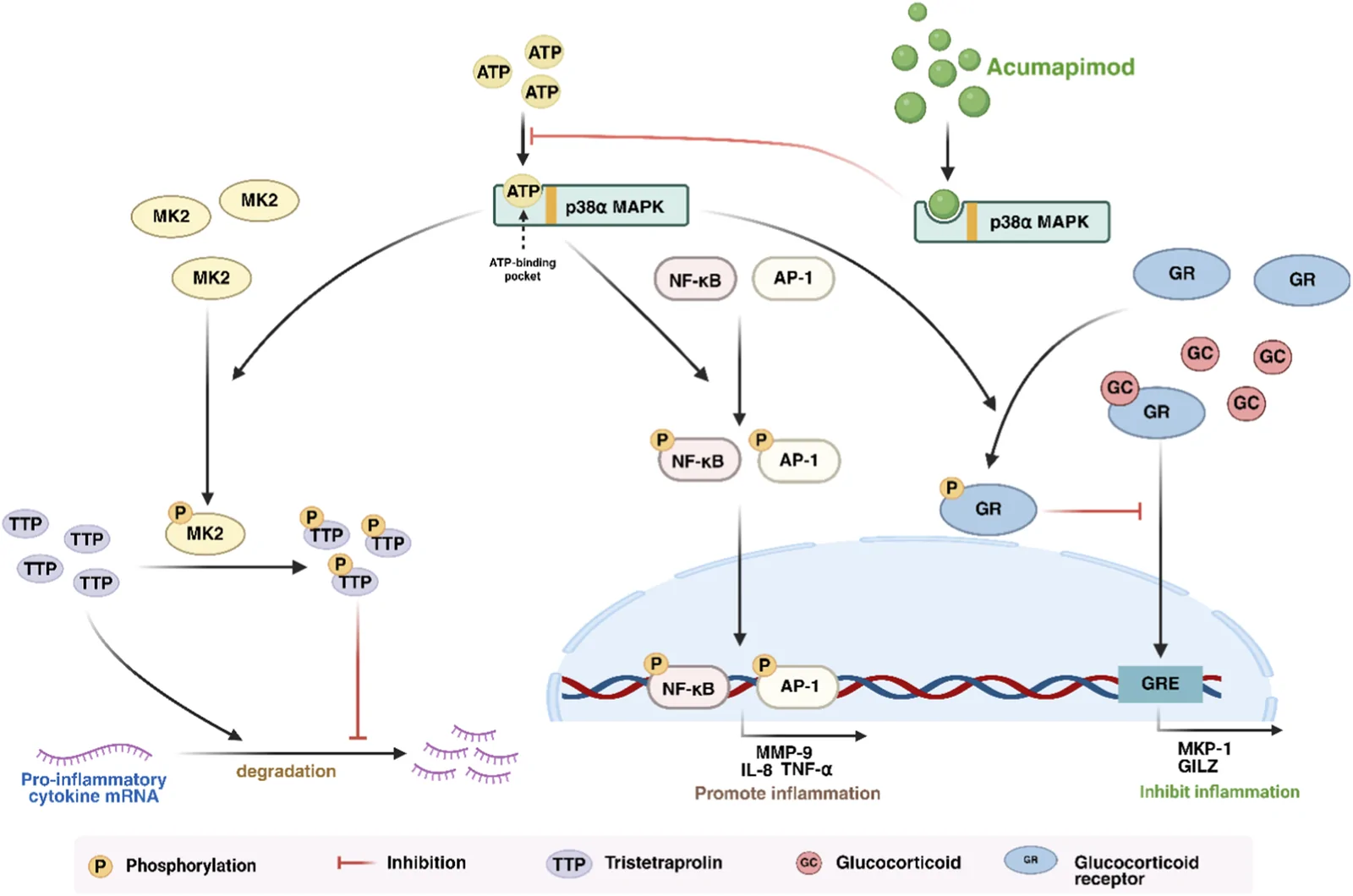
Mechanisms of action of Acumapimod in COPD. Acumapimod inhibits p38α MAPK signaling, suppressing NF-κB/AP-1–mediated inflammatory gene transcription and enhancing TTP-dependent degradation of pro-inflammatory cytokine mRNAs. In addition, it may restore glucocorticoid sensitivity through modulation of GR signaling, thereby reducing airway inflammation in COPD. This figure was enhanced using ChatGPT (GPT-4o) for resolution improvement and noise reduction.

p38 mitogen-activated protein kinase (p38 MAPK) is a central regulator of cellular stress and inflammatory responses. Among its isoforms, p38α plays a key role in the pathogenesis of COPD by promoting airway inflammation, oxidative stress, tissue remodeling, and corticosteroid resistance (Ahmadi et al., 2023). At the transcriptional level, p38α activates downstream kinases MSK1/2, leading to phosphorylation of NF-κB p65 and AP-1 and enhanced transcription of pro-inflammatory genes. At the post-transcriptional level, p38α activates MAPK-activated protein kinase 2 (MK2), which phosphorylates the AU-rich element-binding protein tristetraprolin (TTP), impairing its mRNA-destabilizing function and increasing the stability and translation of inflammatory mediators such as IL-6, IL-8, and TNF-α. In addition, p38α directly phosphorylates the glucocorticoid receptor, thereby inhibiting its nuclear translocation and transcriptional activity and reducing responsiveness to corticosteroids (Pelaia et al., 2021; Ahmadi et al., 2023). Accordingly, p38 MAPK has emerged as an important therapeutic target in COPD, and current clinical development has focused primarily on selective p38α inhibitors and dual p38α/β inhibitors, with CHF6297 and acumapimod representing the leading investigational agents (De Buck et al., 2015) (Figure 2).

Acumapimod is an orally administered selective p38α/β MAPK inhibitor developed for the treatment of acute exacerbations of COPD (AECOPD). By selectively inhibiting p38 MAPK phosphorylation, Acumapimod suppresses the production of multiple pro-inflammatory mediators, including TNF-α, IL-1β, IL-6, and CXCL8, thereby attenuating neutrophil-driven airway inflammation. Preclinical studies exhibited that p38 MAPK inhibition effectively reduced inflammatory cell recruitment and cytokine release in cigarette smoke–induced airway inflammation models, supporting its therapeutic potential in COPD. Furthermore, pharmacodynamic studies showed that Acumapimod rapidly penetrates pulmonary tissues and achieves sustained target engagement, providing a mechanistic basis for its anti-inflammatory effects (Strâmbu et al., 2019; Agyemang et al., 2021). In a phase II clinical trial, acumapimod was evaluated in patients with moderate-to-severe AECOPD and showed clinically meaningful improvements in lung function. At Day 8, treatment with Acumapimod 75 mg resulted in a placebo-adjusted increase of 152 mL in FEV_1_ (P < 0.001). Post hoc analyses further showed sustained improvements in FEV_1_ over 14 days compared with both placebo and prednisone. In parallel, Acumapimod produced a 72% reduction in high-sensitivity C-reactive protein (hsCRP), comparable to that observed with prednisone (74%), indicating potent anti-inflammatory activity. Importantly, improvements in lung function were observed as early as Day 3 and were maintained throughout the treatment period. However, in the same phase II trial, the primary endpoint at Day 10 was not met. The 75 mg repeat-dose group exhibited a placebo-adjusted FEV_1_ difference of 124 mL (95% confidence interval [–16, 263]; p = 0.082), which did not achieve statistical significance. This outcome was attributed primarily to the dosing regimen. Given the 30- to 35-h half-life of acumapimod, administration on Days 1 and 6 likely failed to sustain sufficient plasma concentrations through Day 10, a hypothesis corroborated by the significant efficacy observed at Day 8. Of note, the early and robust improvement in FEV_1_ at Day 8, together with the sustained benefit over 14 days in *post hoc* analyses, reinforces the compound’s biological activity. Collectively, these findings suggest that although the chosen schedule was suboptimal for the primary timepoint, the overall clinical and biomarker data—including the substantial reduction in hsCRP—provide a strong rationale for future trials with optimized regimens, such as alternate-day dosing, to fully realize the therapeutic potential of acumapimod in AECOPD (Strâmbu et al., 2019).

CHF6297 is a highly selective p38α MAPK inhibitor formulated as a dry powder for inhalation, designed to achieve potent local anti-inflammatory activity with minimal systemic exposure (Ahmadi et al., 2023; Martucci et al., 2024). Previous studies have reported that CHF6297 possesses potent anti-inflammatory activity in both cellular and animal models. In lipopolysaccharide (LPS)-stimulated rat peripheral blood mononuclear cells and cigarette smoke extract (CSE)-stimulated human airway smooth muscle cells, CHF6297 inhibited TNF-α and IL-8 release with IC_50_ values of 1.4 and 0.79 nM, respectively (Armani et al., 2022; Martucci et al., 2024). *In vivo*, CHF6297 exhibited potent anti-inflammatory activity. In a cigarette smoke–induced pulmonary inflammation model, CHF6297 reduced neutrophil infiltration in bronchoalveolar lavage fluid (BALF) by 62%. Furthermore, in an influenza-induced model of COPD exacerbation, CHF6297 at a dose of 0.1 mg/kg almost completely reversed airway neutrophilia. Notably, CHF6297 exhibited synergistic anti-inflammatory effects when combined with budesonide, suggesting its potential utility in the treatment of corticosteroid-resistant airway inflammation (Martucci et al., 2024). Importantly, CHF6297 maintained potent pulmonary anti-inflammatory activity while exhibiting minimal systemic effects, reflecting a favorable lung-restricted profile. Such a property may mitigate the systemic adverse events that have limited the clinical utility of earlier oral p38 MAPK inhibitors (Armani et al., 2022; Martucci et al., 2024). Given the encouraging preclinical profile of CHF6297—including its potent local anti-inflammatory activity, synergistic effects with corticosteroids, and favourable lung-restricted safety margin—the compound was advanced into a first-in-human, randomized, double-blind, placebo-controlled phase I/II trial (NCT02815488). The trial was designed to enroll 118 participants across four modules: single- and multiple-ascending dose evaluations in healthy volunteers for safety and pharmacokinetics; multiple-dose safety and preliminary efficacy in COPD patients; and a lipopolysaccharide challenge in healthy subjects to assess anti-inflammatory activity. However, owing to considerable recruitment difficulties encountered in the fourth module, the trial was prematurely terminated. Consequently, this investigation failed to generate published efficacy data for CHF6297 in the COPD population.

### 3.2 Targeting the Nrf2–Keap1 pathway

The nuclear factor erythroid 2–related factor 2 (Nrf2)–Kelch-like ECH-associated protein 1 (Keap1) axis is a central regulatory pathway governing cellular antioxidant and anti-inflammatory defense mechanisms and represents an important therapeutic target for small-molecule drug development in COPD. Under physiological conditions, Keap1 binds to Nrf2 and promotes its ubiquitination and subsequent proteasomal degradation, thereby maintaining low basal levels of Nrf2 expression (Fratta Pasini et al., 2020; Gao et al., 2025). Chronic exposure to cigarette smoke and environmental pollutants leads to excessive generation of reactive oxygen species (ROS), resulting in dysregulation of the Nrf2 signaling in patients with COPD. Reduced Nrf2 expression, impaired nuclear translocation, and diminished transcriptional activity limit the induction of antioxidant response element (ARE)-dependent cytoprotective genes, such as HO-1 and NQO1. As a result, antioxidant defense mechanisms are weakened, contributing to sustained oxidative stress, chronic inflammation, and progressive tissue injury in COPD (Boutten et al., 2011; Barnes, 2020). Given the central role of Nrf2 dysfunction in the pathogenesis of COPD, pharmacological activation of the Nrf2–Keap1 axis to restore endogenous antioxidant defenses has emerged as an attractive therapeutic strategy. Among the emerging Nrf2-targeted agents, CPUY192018, tussilagone (TUS), and omaveloxolone have been reported to show promising therapeutic efficacy in experimental studies and are currently being actively investigated for the treatment of COPD (Li et al., 2022).

**FIGURE 3:**
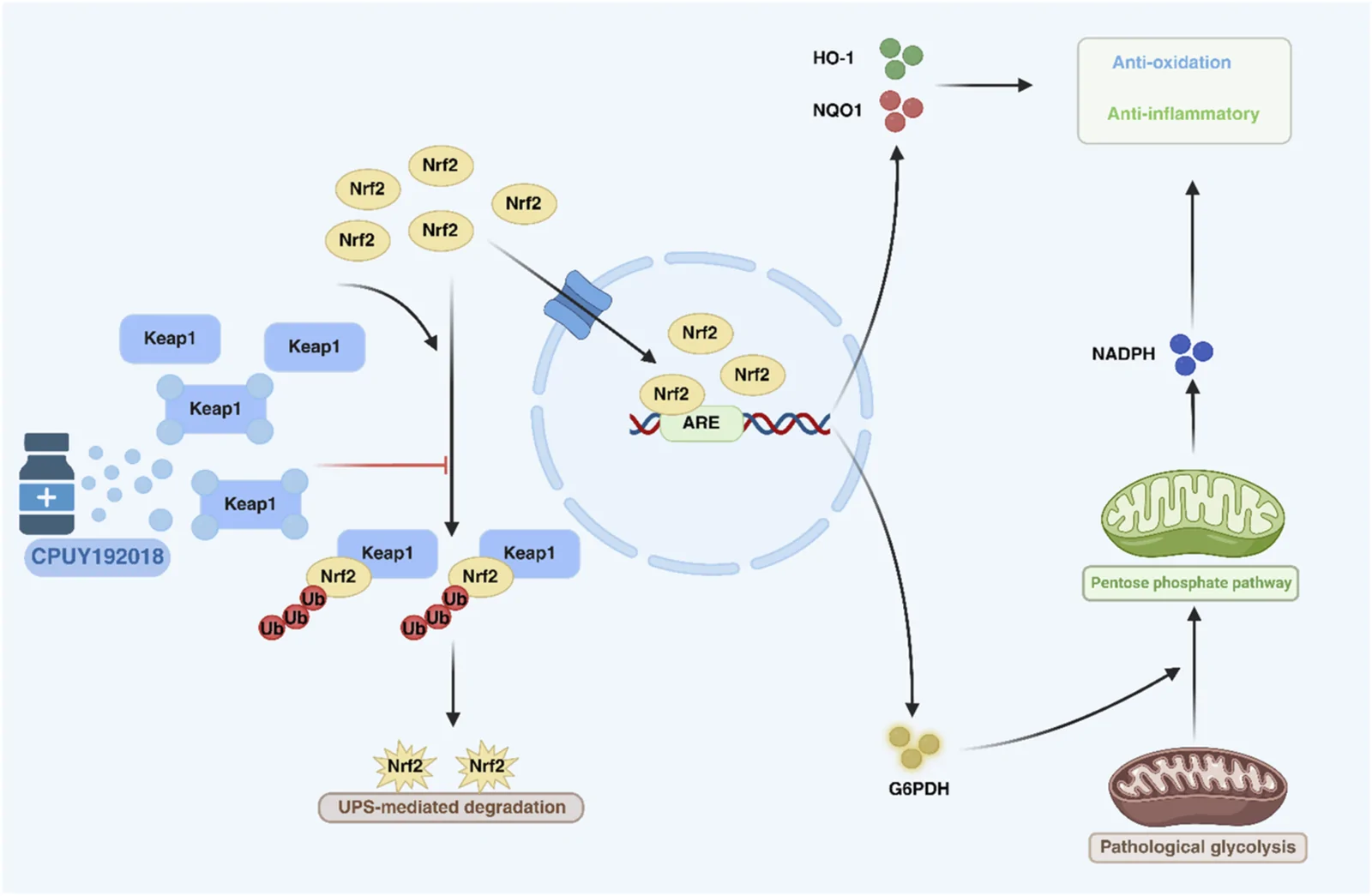
Mechanism of action of CPUY192018 via the Nrf2–Keap1 Pathway. CPUY192018 disrupts the Keap1–Nrf2 interaction, promotes Nrf2 nuclear translocation and ARE-dependent transcription, induces antioxidant enzymes such as HO-1 and NQO1, and reprograms glucose metabolism to exert antioxidant and anti-inflammatory effects. This figure was enhanced using ChatGPT (GPT-4o) for resolution improvement and noise reduction.

CPUY192018 is a small-molecule inhibitor of the Keap1–Nrf2 protein–protein interaction. Based on drug-screening and structure-guided optimization studies, Wang and colleagues reported that CPUY192018 binds with high affinity to the Kelch domain of Keap1, thereby preventing Keap1-mediated sequestration and degradation of Nrf2. This interaction prevents Keap1-mediated ubiquitination and proteasomal degradation of Nrf2, thereby activating Nrf2-dependent ARE signaling and restoring cellular antioxidant defenses (Jiang et al., 2014; Wang et al., 2021). CPUY192018 exerts anti-COPD effects through a dual mechanism involving activation of antioxidant signaling and macrophage metabolic reprogramming. Treatment with CPUY192018 markedly promoted Nrf2 nuclear translocation, leading to an approximately fivefold increase in nuclear Nrf2 levels. This was accompanied by a 3–5-fold upregulation of ARE-dependent antioxidant and detoxification genes, including HO-1, NQO1, and GST. Consequently, antioxidant defense capacity in alveolar macrophages was restored, thereby alleviating oxidative stress–induced injury associated with COPD (Lu et al., 2019; Wang et al., 2021). Furthermore, CPUY192018 dose-dependently induced the expression of glucose-6-phosphate dehydrogenase (G6PD), a key metabolic enzyme regulated downstream of Nrf2. At a concentration of 10 μM, G6PD expression increased by approximately 3–4-fold. Upregulation of G6PD activated the pentose phosphate pathway and enhanced NADPH production, thereby promoting a metabolic shift in macrophages from pathological glycolysis toward mitochondrial oxidative phosphorylation. Through metabolic reprogramming, CPUY192018 not only attenuated persistent inflammatory activation but also restored macrophage phagocytic function, further enhancing host defenses against oxidative stress and inflammatory injury (Lu et al., 2019; Wang et al., 2021; Xu et al., 2024). In a CSC-induced COPD mouse model, treatment with CPUY192018 (20 mg/kg) markedly suppressed pulmonary inflammation, reducing BALF neutrophils by 66.7% and lowering lung IFN-γ, IL-5, IL-6, and TNF-α levels by over 60%. Moreover, CPUY192018 restored macrophage phagocytic capacity from 48% to 89% and attenuated airway inflammation, emphysematous destruction, and pathological lung remodeling (Wang et al., 2021) (Figure 3). Collectively, CPUY192018 has shown potential as a Nrf2 activator for the treatment of COPD in preclinical studies; however, the compound has not yet entered human clinical trials, and its clinical efficacy and safety remain to be validated in future investigations.

Using drug screening, target validation, and experimental studies in cellular and animal models, TUS was shown to exert potent antioxidant activity by directly targeting the Cys434 residue of Keap1. Covalent modification of Keap1 disrupted Nrf2 ubiquitination and degradation, thereby promoting Nrf2 nuclear translocation and activation of the Nrf2–ARE signaling pathway. Treatment with TUS (1.25–10 μM) increased GCLM and NQO1 mRNA expression by up to 2.2-fold and 3.2-fold, respectively, while enhancing GSH and NAD(P)H quinone dehydrogenase activities by 3.4-fold and 2.8-fold. These effects effectively reduced intracellular ROS accumulation and alleviated oxidative stress associated with COPD (Xu et al., 2022; Xu et al., 2026). Moreover, activation of the Nrf2 signaling further suppressed inflammatory responses, resulting in 48.6% and 43.8% reductions in iNOS and COX-2 expression, respectively. TUS also inhibited NLRP3 inflammasome activation by blocking the assembly of the NLRP3–ASC–caspase-1 complex, thereby preventing the propagation of downstream inflammatory cascades (Sun et al., 2024; Xu et al., 2026). In a cigarette smoke–induced COPD mouse model, treatment with TUS (20 mg/kg, intraperitoneally) markedly attenuated pulmonary inflammation, reducing BALF neutrophil and macrophage infiltration by approximately 65% and 55%, respectively. Furthermore, pulmonary levels of the pro-inflammatory cytokines TNF-α and IL-1β were reduced by 68.1% and 57.7%, respectively, indicating potent suppression of cigarette smoke–induced inflammatory responses (Xu et al., 2026). Histopathological analyses further indicated that TUS markedly ameliorated cigarette smoke–induced lung injury, reducing alveolar destruction by approximately 70%, improving alveolar structural damage by more than 80%, and decreasing airway collagen deposition and fibrosis by approximately 60% and 58%, respectively. Pulmonary inflammation scores were reduced by approximately 65%. Moreover, TUS suppressed MUC5AC overproduction by approximately 59% and restored surfactant protein D (SP-D) levels to nearly 85% of those observed in healthy controls, collectively indicating substantial reversal of cigarette smoke–induced pulmonary pathology and functional impairment (Xu et al., 2026). Overall, although TUS has shown potential for the treatment of COPD in preclinical studies, its efficacy and safety have not yet been validated in humans.

Omaveloxolone is a synthetic triterpenoid Nrf2 activator that enhances cellular antioxidant and anti-inflammatory defenses by relieving Keap1-mediated repression of Nrf2, thereby promoting Nrf2 nuclear translocation and activating the transcription of ARE-dependent genes (Probst et al., 2015; Hao et al., 2024). Evidence from a PM2.5-induced COPD rat model indicates that omaveloxolone activates the Nrf2/HO-1 signaling pathway in a dose-dependent manner, while also inhibiting aberrant NF-κB and IFN-γ production. After 1 week of treatment, pulmonary Nrf2 and HO-1 expression levels in the 3 mg/kg group increased by approximately 2.8-fold and 2.5-fold, respectively, compared with the model group, whereas NF-κB and IFN-γ levels decreased by approximately 62% and 58%, respectively. In addition, serum nitric oxide (NO) levels were restored to 78% of normal values, BALF neutrophil infiltration was reduced by more than 60%, and both oxidative lung injury and alveolar structural destruction were markedly attenuated (Niu et al., 2024). In a PM2.5-induced human bronchial epithelial cell model, omaveloxolone also exhibited significant cytoprotective effects. Treatment reversed the PM2.5-induced reduction in cell viability, restored impaired Nrf2/HO-1 signaling, and downregulated the expression of airway remodeling-associated factors, including transforming growth factor-β1 (TGF-β1), matrix metalloproteinase-9 (MMP-9), and fibronectin (FN). These findings suggest that omaveloxolone not only alleviates oxidative stress and inflammatory responses but may also delay COPD progression by attenuating airway remodeling (Sun et al., 2020; Niu et al., 2024). Omaveloxolone has been approved by the FDA for Friedreich ataxia; however, its application in COPD is still limited to preclinical investigations. While preliminary data suggest biological activity, the lack of well-controlled, adequately powered clinical trials means that its benefit–risk profile in COPD patients remains to be established.

### 3.3 Targeting PDE4 in COPD

The phosphodiesterase-4 (PDE4) family is widely expressed in neutrophils, macrophages, T lymphocytes, and airway epithelial cells. By hydrolyzing intracellular cAMP, PDE4 attenuates cAMP/PKA-mediated anti-inflammatory signaling, leading to activation of pro-inflammatory transcription factors such as NF-κB and increased production of inflammatory mediators, thereby amplifying airway inflammation (Gao et al., 2025; Kelly et al., 2025). In contrast, the phosphodiesterase-3 (PDE3) family is predominantly expressed in airway and vascular smooth muscle cells and primarily regulates airway smooth muscle tone. Both PDE3 and PDE4 are established therapeutic targets in COPD (Anzueto et al., 2023; Li et al., 2025). Roflumilast and ensifentrine have been approved for COPD treatment (Martinez et al., 2015; Li et al., 2025). However, the clinical use of currently available PDE inhibitors remains limited by tolerability concerns, particularly the gastrointestinal adverse effects associated with roflumilast.

**FIGURE 4:**
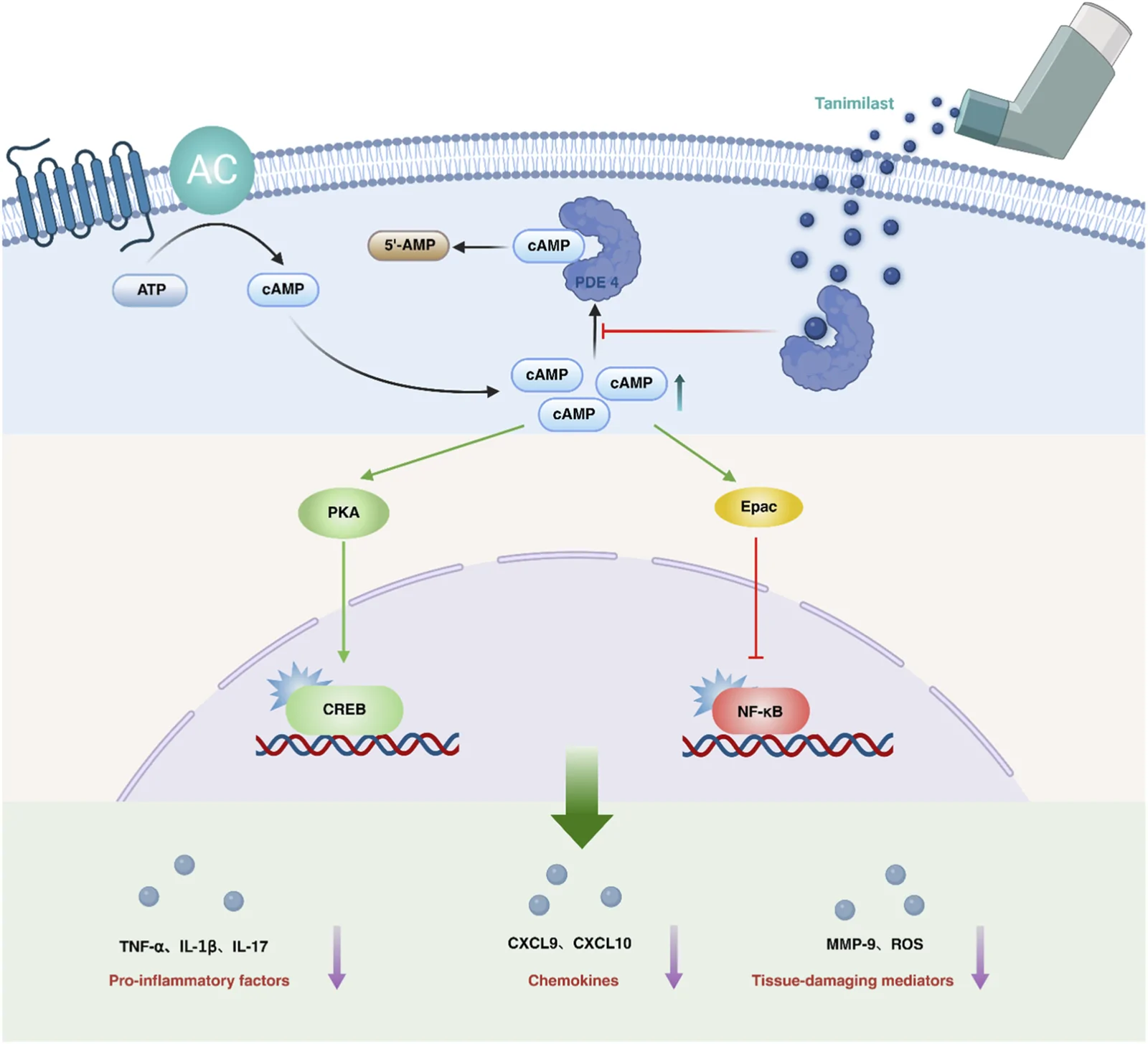
Mechanism of action of Tanimilast in COPD. Tanimilast inhibits PDE4, thereby increasing intracellular cAMP levels. Elevated cAMP activates the PKA–CREB and epac pathways, promotes anti-inflammatory gene transcription, suppresses NF-κB signaling, and reduces the release of pro-inflammatory mediators, chemokines, and tissue-damaging factors. This figure was enhanced using ChatGPT (GPT-4o) for resolution improvement and noise reduction.

Studies have reported that tanimilast possesses exceptionally high inhibitory potency toward PDE4, with an IC_50_ of 0.026 nM compared with 0.26 nM for roflumilast, indicating an approximately 10-fold increase in enzymatic potency (Moretto et al., 2015). Through inhibition of PDE4-mediated cAMP hydrolysis, tanimilast increases intracellular cAMP concentrations, leading to activation of downstream signaling pathways such as CREB and consequent suppression of inflammatory responses, while simultaneously promoting tissue-protective effects (Facchinetti et al., 2021). In cellular and *ex vivo* lung tissue studies, tanimilast exhibited potent anti-inflammatory and anti-remodeling effects. A study using alveolar macrophages from COPD patients found that tanimilast inhibited TNF-α and IL-6 release with EC_50_ values of 0.02 nM and 0.01 nM, respectively, and that its inhibitory potency was substantially greater than that of roflumilast (Lea et al., 2019). Moreover, precision-cut lung slices from explanted COPD lungs treated with 0.1 μM tanimilast showed reductions in MUC5AC and MUC5B secretion of 50.6% and 31%, respectively. Concurrently, the release of MCP-1 was decreased by 44%, indicating that tanimilast may exert beneficial effects beyond anti-inflammatory activity by attenuating mucus hypersecretion and airway remodeling, two hallmark pathological features of COPD (Facchinetti et al., 2021). Beyond macrophage-directed effects, tanimilast also modulates neutrophil-driven inflammation, a key contributor to COPD pathogenesis. Schioppa and colleagues reported that tanimilast inhibited NE and MPO release with IC_50_ values of 49.2 and 48.8 nM, respectively. Notably, significant suppression of MMP activity and neutrophil-derived protease release was observed even at nanomolar concentrations, indicating potent inhibition of neutrophil effector functions. In addition, by enhancing cAMP-dependent signaling, tanimilast preserved neutrophil homeostasis and regulated activation status, thereby reducing protease-mediated tissue injury and oxidative stress and limiting secondary inflammatory damage (Schioppa et al., 2022). The phase IIb PIONEER study further showed the clinical efficacy of tanimilast in patients with COPD with a chronic bronchitis phenotype and baseline blood eosinophil counts ≥150 cells/μL. Twice-daily administration of tanimilast at 800 and 1,600 μg was associated with 49% and 73% reductions in exacerbation risk, respectively. Importantly, treatment-related adverse events were reported in only 3.1%–5.3% of patients, a lower incidence than that observed in the placebo group (7.3%). In contrast to conventional PDE4 inhibitors such as Roflumilast, Tanimilast was not associated with the typical gastrointestinal adverse events, including nausea and diarrhea, suggesting an improved safety and tolerability profile (Singh et al., 2020; Tousi et al., 2025) (Figure 4).

Despite favorable tolerability and the lack of unexpected safety findings, the phase III PILASTER trial of Tanimilast did not achieve its primary endpoint: the drug failed to produce a significant reduction in annual moderate-to-severe exacerbation rates when added to triple therapy in patients with moderate-to-severe COPD. Similarly, no significant treatment effects were detected for key secondary outcomes, including time to first exacerbation, severe exacerbation rate, lung function, or patient-reported outcomes. As a consequence of these negative efficacy results, Chiesi Group decided in November 2025 to discontinue all further clinical development of Tanimilast for COPD and asthma.

## 4 Mesenchymal stem cells

Mesenchymal stem cells (MSCs) are the most extensively studied cell therapy for COPD and have substantial translational potential (Calzetta et al., 2022). The therapeutic effects of MSCs are primarily attributed to their potent immunomodulatory, antioxidant, and tissue-reparative properties. Through paracrine signaling and cell–cell interactions, MSCs promote macrophage polarization toward the anti-inflammatory M2 phenotype, suppress aberrant activation of neutrophils and T lymphocytes, and re-establish the balance between pro-inflammatory and anti-inflammatory cytokines, thereby alleviating persistent airway inflammation and immune dysregulation (Armitage et al., 2021; Glassberg et al., 2021). MSCs also alleviate oxidative stress by enhancing Nrf2-mediated antioxidant defenses, thereby reducing oxidative damage caused by cigarette smoke and LPS (Zhou et al., 2025). In addition, MSCs can differentiate into alveolar epithelial-like cells and secrete reparative factors that stimulate endogenous lung progenitor cells, thereby promoting alveolar regeneration and improving gas exchange (Iwatake et al., 2024). Furthermore, MSCs can transfer functional mitochondria and release extracellular vesicles containing bioactive cargo, thereby restoring cellular bioenergetics, inhibiting apoptosis of injured airway epithelial cells, and facilitating tissue repair (Ridzuan et al., 2021; Melo et al., 2023).

### 4.1 Remestemcel-L

Remestemcel-L is an allogeneic bone marrow-derived mesenchymal stem cell (BMSC) product developed for moderate-to-severe COPD with a highly inflammatory phenotype. Its therapeutic effects are mediated primarily through paracrine and immunomodulatory mechanisms rather than direct differentiation into lung tissue cells, targeting chronic inflammation, oxidative injury, and airway and alveolar remodeling (Kennelly et al., 2016). Research by Helen Kennelly and colleagues shows that BMSCs exert therapeutic effects through multiple mechanisms, including NF-κB pathway inhibition and secretion of HGF, VEGF, and regulatory TGF-β. BMSC treatment reduced the levels of pro-inflammatory mediators, such as TNF-α, IL-6, and CXCL8, thereby attenuating pulmonary neutrophil infiltration and macrophage activation (Kennelly et al., 2016; Weiss et al., 2021). In addition, BMSCs downregulated the expression of MUC5AC and MUC5B, leading to reduced airway mucus hypersecretion. They also decreased ROS production and Caspase-3 activity, thereby mitigating oxidative stress and apoptosis. It has been reported that BMSC exosomes exert therapeutic effects in COPD models through the miR-30b/Wnt5a pathway. Exosomal delivery of miR-30b increased its expression by approximately threefold, suppressed Wnt5a expression by 61%–68%, and reduced CSE-induced endothelial cell apoptosis from 38.2% to 13.5%. In COPD models, BMSC-derived exosomes resulted in improvement in alveolar architecture and attenuated emphysematous changes, highlighting their potential as an acellular therapeutic strategy for COPD (Weiss et al., 2013; Song et al., 2025).

In a phase I/II randomized, placebo-controlled trial of Remestemcel-L in patients with COPD, the primary analysis demonstrated a favorable safety profile but failed to detect significant efficacy on pulmonary function in the overall cohort. Nevertheless, exploratory *post hoc* analyses revealed that treatment effects were contingent upon baseline inflammatory status, with meaningful improvements in lung function and exercise capacity observed exclusively in patients presenting with elevated C-reactive protein levels. These findings imply that the therapeutic activity of Remestemcel-L may be largely confined to a phenotypically distinct subgroup characterized by heightened systemic inflammation. Importantly, even among these responders, the clinical gains appeared to be short-lived; despite partial persistence over the 2-year observation period, the overall trajectory did not support a sustained disease-modifying potential (Weiss et al., 2021).

### 4.2 hUC-MSCs

Human umbilical cord–derived mesenchymal stem cells (hUC-MSCs) are considered one of the most promising stem cell–based therapies for COPD owing to their non-invasive procurement, low immunogenicity, high proliferative potential, and potent paracrine activity (Laiman et al., 2022; Iwatake et al., 2024; Ridzuan et al., 2021). It has been suggested that hUC-MSCs exert therapeutic effects via dual signaling pathways in a murine emphysema model. Specifically, hUC-MSCs treatment inhibited the NF-κB inflammatory pathway, resulting in a 40%–60% reduction in TNF-α levels, a 58%–62% decrease in IL-1β, a 65%–72% reduction in pulmonary neutrophil infiltration, and over a 50% decrease in alveolar macrophage activation, thereby attenuating the pulmonary inflammatory cascade (Laiman et al., 2022). In addition, hUC-MSCs regulated the Hippo–YAP pathway, increasing the p-YAP/YAP protein ratio by more than twofold compared with the COPD model group. By restoring YAP phosphorylation and inhibiting its nuclear translocation, hUC-MSCs not only suppressed excessive inflammatory responses but also promoted the proliferation, differentiation, and self-renewal of alveolar type II epithelial cells, thereby facilitating alveolar repair and reversing structural lung damage (Laiman et al., 2022). Previous work indicated that human umbilical cord mesenchymal stem cell-derived extracellular vesicles (hUC-MSC-EVs) recapitulate the therapeutic effects of their parent cells in a cigarette smoke–induced rat model of COPD. In addition, treatment enhanced alveolar architecture, reducing the MLI from 108.7 to 62.5 μm. These findings support the potential of hUC-MSC-EVs as a promising cell-free regenerative therapy for COPD (Ridzuan et al., 2021).

Noridzzaida Ridzuan and colleagues developed engineered human umbilical cord–derived mesenchymal stem cells (dUC-MSCs) with enhanced cell-adhesive properties, resulting in prolonged pulmonary retention of up to 24 h and an over 80% increase in lung retention compared with conventional hUC-MSCs. In animal models of emphysema, dUC-MSCs were associated with enhanced macrophage polarization toward the anti-inflammatory M2 phenotype, as reflected by a 47% increase in M2 macrophage proportion relative to the emphysema model group and a 41% increase relative to conventional hUC-MSCs treatment. These effects were associated with enhanced immunomodulatory activity and improved lung tissue repair (Iwatake et al., 2024). Regarding pulmonary structural restoration, dUC-MSC treatment increased total alveolar area by 35%, ameliorated alveolar wall collapse, and reduced the MLI by 42% compared with the model group. Concurrently, anti-inflammatory efficacy was improved, with pulmonary IL-1β and IL-6 expression reduced by 68% and 62%, respectively, reflecting a comprehensive augmentation of therapeutic outcomes (Iwatake et al., 2024).

The translation of hUC-MSC-based therapies from bench to bedside is hindered by several formidable challenges. Foremost, mesenchymal stem cells derived from disparate tissue sources exhibit marked heterogeneity in proliferative capacity, differentiation potential, and secretory profiles, which compromises cross-study comparability and impedes the development of standardized manufacturing and quality control protocols (Mendicino et al., 2014; Samsonraj et al., 2017; Pittenger et al., 2019). Secondly, the fundamental mechanisms underpinning their therapeutic actions—including immunomodulation, anti-inflammatory effects, and tissue regeneration—remain incompletely characterized, thereby constraining the rational design and optimization of clinical regimens (Galipeau and Sensébé, 2018; Levy et al., 2020). Moreover, the majority of available clinical trials are limited by small sample sizes, and a number of favorable findings derive from *post hoc* analyses, collectively rendering the current body of evidence insufficient (Thompson et al., 2020). Consequently, the clinical utility of hUC-MSCs mandates rigorous validation through well-powered, methodologically robust.

### 4.3 AD-MSCs

Adipose-derived mesenchymal stromal cells (AD-MSCs) represent a highly promising MSC source for COPD treatment, attributable to their high tissue yield, accessible harvest procedure, and potent immunomodulatory and regenerative capacities (Zhang et al., 2022). Substantial preclinical evidence substantiates the therapeutic potential of AD-MSCs in emphysema models (Arruda de Faria et al., 2021). Hong and colleagues demonstrated that pioglitazone preconditioning significantly enhanced the efficacy of AD-MSCs in smoke-induced emphysema, as evidenced by a reduction in mean linear intercept from 80.5 ± 3.2 μm to 78.1 ± 2.5 μm, suggesting that pharmacological modulation may augment their reparative capacity. Nevertheless, a pivotal consideration for autologous transplantation is whether AD-MSCs derived from COPD patients retain functional competence. Addressing this, Martín Medina and colleagues compared COPD- and non-COPD-derived AD-MSCs and found that, despite impaired *in vitro* migratory responses to VEGF and cigarette smoke, the COPD-derived cells were equally effective in mitigating elastase-induced lung emphysema in a murine model. These findings collectively support the feasibility of autologous AD-MSC-based therapy in COPD, indicating that disease-origin cells maintain therapeutic efficacy even though specific migratory pathways are compromised.

The translation of AD-MSC therapies from preclinical studies to clinical application is steadily advancing, as evidenced by a phase I open-label trial that established favorable safety and feasibility profiles in patients with moderate-to-severe COPD and combined pulmonary fibrosis and emphysema (CPFE), although no significant mean changes in pulmonary function parameters (FVC, FEV1, TLC, TLco) were observed—an outcome that nonetheless warranted progression to phase II testing (Tzilas et al., 2015; Cheng et al., 2017). Concurrently, the therapeutic landscape is being further explored through multiple ongoing or completed clinical investigations, including the phase III RESPIRE trial, a randomized, multicenter, double-blind, placebo-controlled study designed to assess the efficacy of AD-MSC therapy in improving FEV1 in COPD patients (Zhang et al., 2022). Additional approaches involve the use of autologous adipose-derived stromal vascular fraction (AD-cSVF), harvested via microcannula from subcutaneous adipose tissue and administered intravenously, for chronic lung disorders, as well as the combinatorial strategy of AD-MSCs with activated platelet-rich plasma (PRP), which is currently under investigation as a potential therapeutic regimen. Collectively, these clinical endeavors underscore the progressive momentum toward establishing AD-MSC-based interventions as a viable clinical option for COPD and related pulmonary diseases.

Encouraging as the preclinical and early clinical data may be, the broad clinical adoption of AD-MSC therapy in COPD is constrained by several major challenges. Notable among these are the substantial transcriptomic alterations in COPD-derived AD-MSCs—particularly those involving extracellular matrix remodeling—which may compromise cellular homing, engraftment, and therapeutic outcome (Arruda de Faria et al., 2021; Kruk et al., 2023). Standardization of isolation, expansion, and quality-control procedures is essential to guarantee product uniformity across batches and institutions. Moreover, the optimal route of administration, dosage, and patient selection criteria await definitive elucidation through adequately powered, randomized controlled trials (Cheng et al., 2017).

## 5 Conclusions and future perspectives

COPD is recognized as the third leading cause of mortality worldwide. Current pharmacological management predominantly centers on bronchodilators and inhaled corticosteroids (Cazzola et al., 2025). Although these interventions effectively ameliorate symptomatic manifestations and curtail the frequency of acute exacerbations, their clinical utility remains circumscribed by several notable limitations, including an elevated risk of pneumonia, potential cardiovascular sequelae, corticosteroid insensitivity, and pronounced inter-phenotypic variability in therapeutic responsiveness (van Geffen et al., 2023). More fundamentally, these conventional approaches are incapable of arresting or reversing the progressive decline in lung function. In recent years, progressively refined insights into the pathogenic mechanisms underlying COPD have catalyzed the development and clinical translation of precision-oriented therapeutic strategies (Agusti and Faner, 2026) (Table 1).

**TABLE 1:** Targeted therapies for COPD.

Potential agents	Target	Mechanism	Preclinical evidence	Clinical development status	References
Tezepelumab	TSLP	Inhibiting TSLP upstream alarm signal cascade	Reducing neutrophil/eosinophil recruitment	Phase II COURSE trial completed; global phase III trials ongoing	Verstraete et al. (2017); Singh et al. (2025a) ; Singh et al. (2025c); Lugogo et al. (2026)
Itepekimab	IL-33	Partial blockade of IL-33/ST2 signaling	Attenuating mucus overproduction and lung fibrosis	Phase II positive; two phase III trials with conflicting primary endpoints, regulatory outlook uncertain	Rabe et al. (2021) ; Rabe et al. (2024)
Tozorakimab	IL-33	Complete suppression of IL-33/ST2 and RAGE/EGFR pathways	Improving alveolar destruction and smooth muscle thickening	Phase I/IIa positive; phase III MIRANDA met primary exacerbation endpoint	England et al. (2023); Reid et al. (2024); Singh et al. (2025b)
Astegolimab	ST2 receptor	Blocking IL-33 binding to ST2 receptor	Reducing type 2 inflammatory cell infiltration	Phase II improved lung function; phase III ALIENTO/ARNASA only secondary endpoints significant	Yousuf et al. (2022); Kelsen et al. (2025); Papi et al. (2026)
Acumapimod	p38 MAPK	Blocking p38-mediated NF-κB inflammatory transcription	Reducing TNF-α, IL-6 in smoke-induced lung models	Phase II transient FEV_1_ improvement; day 10 primary endpoint not reached, dosing suboptimal	Strâmbu et al. (2019)
CHF6297	p38 MAPK	Inhaling p38 MAPK inhibitor reducing inflammatory signaling	Synergistic anti-inflammation with inhaled steroids	Phase I/II terminated due to recruitment failure; no clinical efficacy data released	Martucci et al. (2024)
CPUY192018	Nrf2–Keap1	Disrupting Keap1-Nrf2 interaction, stabilizing Nrf2 and activating antioxidant genes	Reprogramming macrophage metabolism, alleviates oxidative injury	no human clinical trials initiated	Wang et al. (2021)
Tussilagone	Nrf2–Keap1	Activating Nrf2 signaling and enhances antioxidant defense	Increasing antioxidant gene expression and reduced ROS-mediated injury	no human clinical trials initiated	Xu et al. (2026)
Omaveloxolone	Nrf2–Keap1	Synthesizing triterpenoid Nrf2 agonist	Enhancing antioxidant responses and reduced oxidative stress	FDA approved for Friedreich ataxia; COPD applications remain preclinical	Niu et al. (2024)
Tanimilast	PDE4	Ultra-potent inhaled PDE4 inhibitor	Suppressing mucus secretion and neutrophil protease release	Phase IIb completed; phase III PILASTER failed to meet primary endpoint	Singh et al. (2020); Facchinetti et al. (2021); Tousi et al. (2025)
Remestemcel-L	Inflammatory microenvironment	Paracrine inhibition of NF-κB, secretion of HGF/TGF-β	Reducing mucus hypersecretion and endothelial apoptosis	Phase I/II safe; only high CRP subgroups show transient lung function benefits	Kennelly et al. (2016); Weiss et al. (2021)
hUC-MSCs	Inflammatory microenvironment	Dual anti-inflammation; alveolar epithelial repair	Improving MLI index and M2 macrophage polarization	Mainly phase I/II studies; efficacy remains unconfirmed	Laiman et al. (2022)
AD-MSCs	Inflammatory microenvironment	Promoting lung repair through immunomodulation, antioxidant effects, and paracrine signaling	Reducing alveolar destruction and inflammatory infiltration in emphysema models	Phase I completed; phase III RESPIRE trial ongoing	Zhang et al. (2022)

In summary, the prevailing clinical evidentiary landscape for precision medicine in COPD unequivocally demonstrates that dupilumab and mepolizumab remain the sole two classes of targeted biologics for which robust Phase III data have been generated and regulatory approval has been secured (Choi, 2026). By contrast, other biologic agents—notably those directed against the TSLP and IL-33/ST2 signaling axes—along with an overwhelming majority of non-biological small-molecule compounds (including p38 MAPK inhibitors, PDE4 inhibitors, and Nrf2 activators) have consistently failed to reproduce their early-phase positive signals in Phase III trials (Kistemaker and Gosens, 2022). This scenario starkly underscores the marked discordance between mechanistic understanding at the preclinical level and its successful translation into clinical practice in COPD drug development. Collectively, these setbacks imply that intrinsic pathobiological heterogeneity of the disease, the limited extrapolative capacity of animal models to the clinical setting, suboptimal sensitivity of selected efficacy endpoints, and imperfectly optimized dosing schedules may collectively represent pivotal impediments that preclude the translation of early positive signals into substantiated clinical benefit (Martinez et al., 2011).

Looking forward, therapeutic strategies for COPD will increasingly pivot toward enhanced individualization and mechanism-based paradigms. Biomarker-informed patient stratification, together with the discovery of novel disease-specific molecular targets, remains a central priority and critical undertaking for future research (Brightling et al., 2025; Singh et al., 2025d). In light of the pronounced inherent heterogeneity of COPD, subsequent trial designs should systematically incorporate inflammatory endotypes or relevant biomarker profiles—including peripheral blood eosinophil counts (BEC), fractional exhaled nitric oxide (FeNO) concentration, or circulating C-reactive protein (CRP) levels—into inclusion criteria, thereby enhancing statistical power and facilitating the accurate identification of potential responder subpopulations (Rabe et al., 2024; Cazzola et al., 2025). Moreover, nucleic acid-based therapeutic modalities—comprising small interfering RNA (siRNA), antisense oligonucleotides (ASOs), and mRNA-based approaches—represent an emerging and highly promising platform with considerable translational potential, capable of selectively silencing pathogenic genes or reinstating functional protein expression (Wang et al., 2025; Chen D. et al., 2026). In parallel, extracellular vesicles derived from mesenchymal stem cells, as a cell-free therapeutic alternative, offer the potential to circumvent the intrinsic safety and standardization hurdles inherent to live-cell-based strategies, and thus merit further investigation. Sustained progress in these cutting-edge technologies is anticipated to fundamentally reconfigure the management paradigm for COPD—from a predominantly symptom-oriented approach toward genuine disease modification and regenerative restoration (Lai and Guo, 2024; Roger et al., 2026). Nevertheless, the fruition of this paradigmatic shift will hinge upon a more comprehensive elucidation of COPD pathogenesis, the formulation of more refined patient selection algorithms, and the deployment of more rigorous and optimally designed clinical trials.
